# Assessing the Pregnancy Protective Impact of Scheduled Nonadherence to a Novel Progestin-Only Pill: Protocol for a Prospective, Multicenter, Randomized, Crossover Study

**DOI:** 10.2196/29208

**Published:** 2021-06-08

**Authors:** Alison Edelman, Agnes Hemon, Mitchell Creinin, Pascale Borensztein, Bruno Scherrer, Anna Glasier

**Affiliations:** 1 Department of Obstetrics & Gynecology Oregon Health & Science University Portland, OR United States; 2 Laboratoire HRA Pharma Chatillon France; 3 Department of Obstetrics & Gynecology University of California, Davis Health Sacramento, CA United States; 4 Bruno Scherrer Conseil St Arnoult en Yvelines France; 5 Centre for Reproductive Health University of Edinburgh Edinburgh United Kingdom

**Keywords:** protocol, missed pill, progestin-only pills, contraception, pharmacokinetics

## Abstract

**Background:**

Progestin-only contraceptive pills (POP) are commonly reserved for women with medical comorbidities but in actuality, POPs can be safely used by anyone wanting to prevent pregnancy. This wide safety profile makes them an ideal candidate for being available over the counter without a prescription, but adherence issues may be more common with over-the-counter use. We need a better understanding of the ability of POPs to prevent pregnancy when adherence issues occur in the form of a missed or delayed pill.

**Objective:**

This study aims to determine cervical mucus characteristics following a 6-hour delayed pill intake or after one missed pill as compared to typical daily use of norgestrel 75 mcg.

**Methods:**

This prospective, multicenter, randomized, crossover study assesses the effect of norgestrel 75 mcg (Opill) on cervical mucus and ovarian activity during reported compliant daily use, after a 6-hour delayed intake mid cycle, and after a mid-cycle missed pill. Subject participation will last approximately 4.5 months. We will recruit at 2 US sites: Oregon Health & Science University, Portland, Oregon and University of California Davis Health, Sacramento, California. Reproductive-aged subjects with regular menstrual cycles (21-35 days), BMI <32 kg/m2, and proven ovulation (screening luteal phase progesterone >3 ng/mL [>10 nmol/L]) are eligible to enroll. Participants cannot be at risk for pregnancy during the study period and not use other hormonal methods. Norgestrel 75 mcg will be taken at the same time daily except for one day in each of treatment periods 2 and 3, when the pill will be taken either 6 hours late (delayed pill) or omitted completely (missed pill). Every 3-4 days, we will monitor subjects for follicular activity with transvaginal ultrasound (TVUS) examination, cervical mucus, and blood sampling for ovarian hormones and gonadotropins. Subjects will undergo serial cervical mucus sampling on the days with missed and delayed pill intake at 8 hours after pill intake on the day before the delayed or missed pill, 3 hours following the scheduled time of pill intake if intake was delayed, 6 hours after the scheduled time if intake was omitted, and on the next day 30 minutes before the time of scheduled pill intake. The primary objective of the study is to determine the effect of a delayed or omitted pill intake on cervical mucus characteristics based on a modified Insler score compared to reported daily use.

**Results:**

Our protocol was successfully approved by a central institutional review board (Advarra, Columbia, MD), received ethical approval on March 23, 2018, and was registered with ClinicalTrials.gov (NCT03585712). As of January 2020, the study completed enrollment of 52 subjects. Analyses are pending.

**Conclusions:**

Our protocol was approved by a central review board, and study procedures were successfully executed with completed proposed enrollment.

**Trial Registration:**

ClinicalTrials.gov NCT03585712; https://clinicaltrials.gov/ct2/show/NCT03585712

**International Registered Report Identifier (IRRID):**

DERR1-10.2196/29208

## Introduction

Oral contraceptives are the most widely used hormonal method of contraception in the United States [1]. Progestin-only pills (POP) are commonly prescribed for individuals with contraindications to estrogen, such as those who are breastfeeding or with medical comorbidities. Almost anyone, with or without a comorbidity, can safely use POPs to prevent pregnancy [2]. This wide safety profile makes them an ideal candidate for being available over the counter without a prescription [3-5]. For successful use, users need a full understanding of the ability of POPs to prevent pregnancy when adherence issues occur in the form of a missed or delayed pill. Studies of combined estrogen-progestin pills are reassuring that short lapses in adherence do not put a user at increased risk for pregnancy; however, we lack similar information for low-dose POPs [6]. These studies are essential to applying for over-the-counter status with the US Food and Drug Administration. POPs are a well-established method of contraception and considered an effective method of birth control with a perfect and typical use failure rate of 0.3% and 9%, respectively [7]. Impenetrable cervical mucus is considered the dominant mechanism for pregnancy prevention with POP use [8]. Timely pill intake is thought to be critical for this mechanism of action, but data are not available to assess the temporality of this effect with low-dose POPs, such as norgestrel 75 mcg (active enantiomer, levonorgestrel), the focus of our proposed studies. POPs also suppress ovarian activity, but the extent of suppression is contingent on progestin potency and dose; but again, data linking pharmacokinetic and pharmacodynamic data for a POP containing norgestrel 75 mcg do not exist [9].

Current clinical guidance recommends that taking POPs more than 3 hours late is considered a missed pill due to concern regarding diminishing effects on cervical mucus [10]. However, that may not be case for all POPs; more detailed studies of cervical mucus qualities over the time period of a missed or delayed pill may prove that a 3-hour cutoff is too short and provide POP users with a wider window for maintaining efficacy should they miss taking their pill on time. Our proposed studies, outlined in the protocol described here, have been designed to determine the impact of the POP-containing norgestrel 75 mcg on cervical mucus and ovarian activity as well as levonorgestrel levels during a treatment period of typical and nonperfect adherence.

The primary objective of the study is to determine the effect on cervical mucus characteristics following a 6-hour delayed pill intake or after 1 missed pill as compared to typical daily use of norgestrel 75 mcg (see Box S1 in Multimedia Appendix 1). Our secondary objectives focus on other pharmacodynamic and pharmacokinetic endpoints including ovarian activity and levonorgestrel levels with typical and nonperfect adherence to POPs.

## Methods

### Study Design

**Figure 1:**
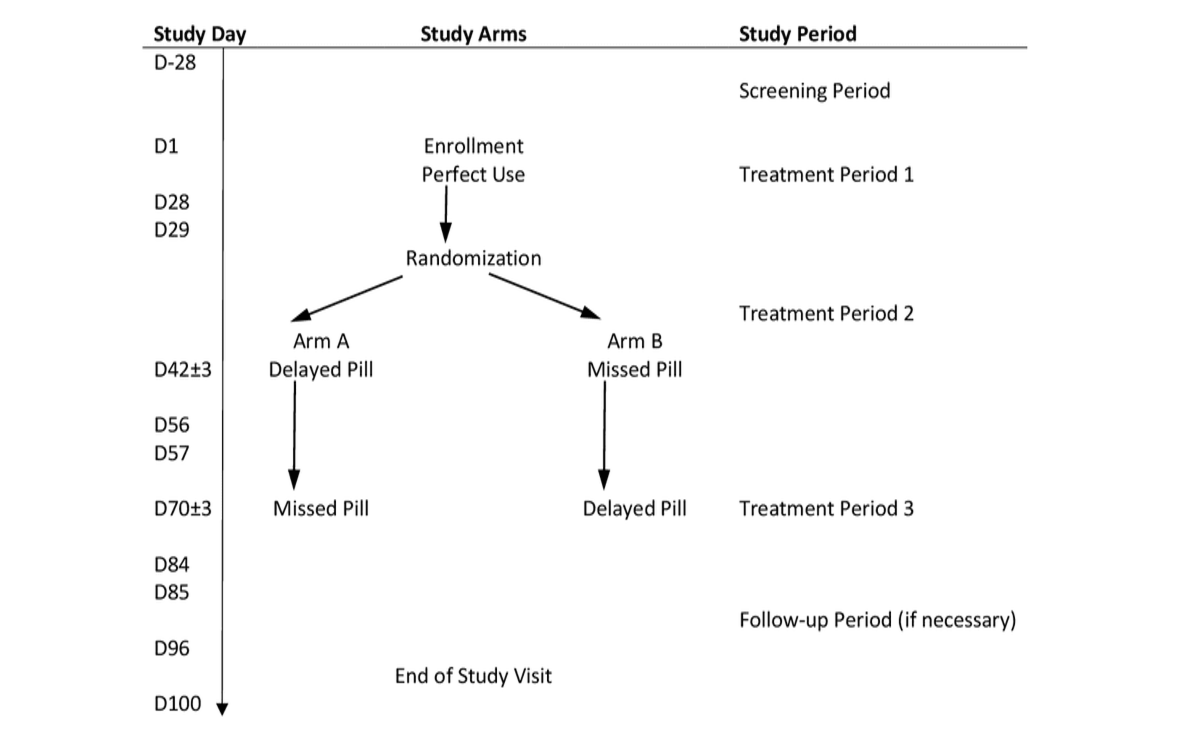
Study design.

This is a prospective, multicenter, randomized, crossover study (Figure 1).

### Study Population

The study population will consist of healthy, reproductive-aged women with regular menstrual cycles.

### Inclusion Criteria

We will recruit healthy subjects aged 18-35 years old with a BMI no greater than 32 kg/m^2^ with regular menstrual cycles (21-35 days) and proven ovulation as documented by a luteal phase progesterone >3 ng/mL (>10 nmol/L) during screening (see Box S2 in Multimedia Appendix 1). Subjects must have an intact uterus and both ovaries. Participants cannot not be at risk for pregnancy during the study period (eg, heterosexually abstinent, using condoms or permanent contraception). Subjects must be willing and able to understand and give informed consent and, in the opinion of the investigator, able to follow all study requirements. Subjects recently postpartum or postabortal must have 1 full menstrual cycle (2 bleeding episodes) and meet the other inclusion criteria prior to enrollment. Subjects previously using intrauterine or noninjectable hormones must have had at least 1 menstrual cycle without the treatment prior to screening. For those having used injectable contraception (depot medroxyprogesterone acetate), their last injection must be at least 9 months before screening.

### Exclusion Criteria

In general, our exclusion criteria include any of the common reasons an individual should not be taking contraception, including pregnancy or anything that would confound or adversely influence our endpoints. A complete list of exclusion criteria can be found in Box S2 in Multimedia Appendix 1.

### Randomization and Allocation Concealment

All subjects will receive the same study drug, norgestrel 75 mcg, during 3 consecutive treatment cycles lasting 28 days each. Treatment cycle 1 will be the planned “perfect use” cycle for all participants with no scheduled delayed or missed pill. We will randomize subjects during the first visit of treatment cycle 2 in a 1:1 ratio with 13 blocks with a block size of 4 for each site, to either Arm A (delayed pill to be done during treatment cycle 2 and missed pill in treatment cycle 3) or Arm B (missed pill in treatment cycle 2 and delayed pill in treatment cycle 3). The centralized study coordinating center will computer-generate the randomization scheme; site-based study staff will not have access to the scheme, but once allocated, both site staff and subjects will know their allocated arm (Figure 1).

### Participant Recruitment and Enrollment

Participants will be recruited from 2 research centers in the United States with competitive recruitment planned between the 2 centers. Subjects will be recruited from clinics serving reproductive-aged women for gynecologic care and reproductive health services (including the clinics’ database) as well as through institutional review board–approved research recruitment efforts (eg, newsletters, in-services, tear ads, social network ads). Referrals from previous or ongoing participants will be accepted. This study’s ClinicalTrials.gov registration number is NCT03585712. Subjects will be selected for the study according to the inclusion and exclusion criteria (see Box S2 in Multimedia Appendix 1), will be provided information about the study and study procedures directly from the study staff, and if eligible and agree to participate, will undergo and sign informed written consent.

### Intervention

Subject participation will be 4.5 months, which includes up to 1 month for screening, 3 separate 28-day treatment cycles, and up to 2 weeks for follow-up and end-of-study processes. All participants will receive the study drug, norgestrel 75 mcg (Opill), in the form of a pill taken orally once daily. The study drug will be provided in boxes containing 1 blister pack of 28 tablets, which corresponds to 28 days of study drug (1 pill per day, single dose). Participants will be provided with 2 boxes at the start of treatment period 1 (one as back up) and 1 box each at the end of treatment periods 1 and 2.

### Sample Size

Since this is an exploratory study with no working hypothesis to confirm or reject, the sample size is not based on a power calculation. This study will screen approximately 70 subjects to achieve at least 45 completed subjects.

### Sample Collection

Serial sampling over the 3 separate 28-day treatment cycles will be necessary to obtain the key pharmacodynamic and pharmacokinetic endpoints for this study: cervical mucus, ovarian follicular activity, gonadotropins and ovarian hormones, and plasma levonorgestrel levels. Subjects will prospectively track any vaginal bleeding experienced.

The integrity of the data for this study is contingent on subject compliance with study drug and procedures. The subjects will be instructed to bring their study drug packs to each visit. Compliance will be assessed by monitoring the study drug present or absent from the medication packs at each visit and at the end of each treatment period and by information recorded prospectively by an electronic diary (daily text messaging to report daily pill intake [yes/no], the time, and bleeding). During treatment cycles 2 and 3 when either the delayed or missed pill will occur, the delayed pill intake will be directly observed by study staff, and the missed pill will be removed from the blister package by the study staff and placed in a small container, labeled, and retained in order to monitor accountability.

**Figure 2:**
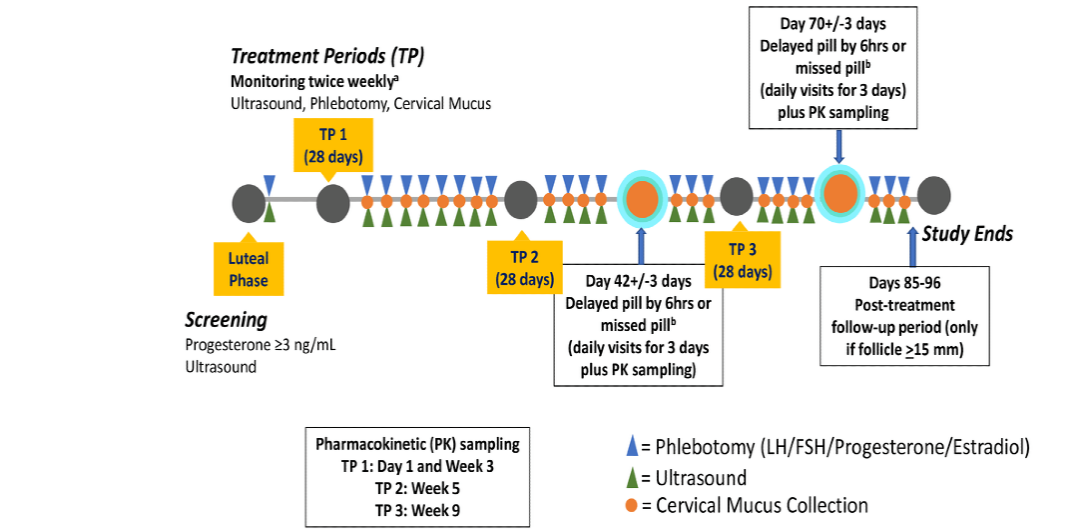
Study Procedures. ^a^If follicle is ≥15mm, then visits are changed to every other day for 3 extra visits; ^b^Three consecutive visits at the time of delayed/missed pill.

Study visits occur approximately every 3-4 days but will increase to every other day when a follicle >15 mm is observed during transvaginal ultrasonography examination (TVUS; for a maximum of 3 visits) and to daily visits around the time of the delayed or missed pill (Figure 2). Cervical mucus, blood sampling, and ovarian monitoring via TVUS will be performed at each visit. Cervical mucus will be collected by aspiration using the SelectMucus Endocervical Aspirator (Cooper Surgical, Trumbull, CT). The cervical mucus assessment will be made by a limited number of trained clinic personnel to limit variability. A centralized in-person training will be organized to maintain a high level of consistency for these evaluations. The cervical mucus sampling will be performed prior to the TVUS examination. We will utilize the modified Insler score to evaluate cervical mucus samples based on consistency (viscosity), ferning, spinnbarkeit, and cellularity, each scored on a 4-point scale (0-3; see Box S3 in Multimedia Appendix 1) [11]. Cervical mucus is considered protective for pregnancy when the total score is ≤4.

TVUS examinations will be performed by licensed and trained study personnel using a transducer of at least 5 mHz. Standard measurements will include documentation of the largest follicle’s maximum measurements in 3 dimensions and average size if it is ≥10 mm, postovulatory image, and any ovarian or follicular abnormalities. A cross-sectional picture of each ovary, even if no follicles >10 mm are present, will also be taken. A postovulatory image will be defined as the abrupt disappearance of the follicle-like structure or reduction of size in the leading follicle by >4 mm at 2 consecutive visits or visualization of a hemorrhagic corpus luteum. Determination of ovarian status will be categorized based on size of follicle and estradiol and progesterone levels according to a modified Hoogland score (Multimedia Appendix 2) by an independent adjudication committee [12,13].

Staff will obtain blood samples for the gonadotropin (luteinizing hormone, follicle-stimulating hormone) and ovarian hormone levels (estradiol, progesterone) during the weekly visits. Levonorgestrel levels will be drawn in treatment cycle 1 on Day 1 and the first visit of week 3, in treatment cycle 2, and in treatment cycle 3.

### Adjudication Committee

An adjudication committee, comprised of experts in cervical mucus assessment and ovarian activity who are not affiliated with either of the study sites, will convene at study end before database lock. This committee will define whether the data collected for a subject were evaluable or relevant and then define ovarian status based on ovarian activity parameters (Multimedia Appendix 2) and the level of conception protection based on cervical mucus (modified Insler score) and ovarian (modified Hoogland score) activity and their temporal relationship.

### Duration of Trial

It was anticipated that the study will be completed in approximately 19 months. As of January 2020, the study completed enrollment of 52 subjects across 2 sites. Analyses are expected to be complete by July 2021.

### Ethical Approval and Dissemination

We utilized a central institutional review board (Advarra, Columbia, MD) and received ethical approval on March 23, 2018. The study was registered with clinicaltrials.gov (NCT03585712) prior to enrollment of the first participant. We plan to present the data at international conferences and publish results in peer-reviewed journals.

### Confidentiality

The information on individual subjects arising from this study is considered confidential and will be transmitted to the sponsor only in a form that will not permit identification of the individual. Identifiable information will remain secure and confidential within the research team. Regulatory and sponsoring agencies may request access to the study records and related medical records of each participating subject; the subject’s identity will remain confidential to the extent permitted by the applicable laws and regulations. The results of the research will be released to public agencies including regulatory agencies, clinical investigators, and research organizations without reference to items identifiable to a particular subject. The results will be published such that the identity of the subjects will not be disclosed and cannot be ascertained. National and international agencies and sponsoring agencies may request access to the medical records of each participating subject, and if requested, the subject’s identity will remain confidential. All records will be kept in a secure storage area with limited access.

### Discontinuations and Withdrawal

A subject is considered to have completed the clinical trial after they have completed 3 separate 28-day treatment periods and the end-of-study visit. Subjects may be discontinued due to an adverse event (AE) or serious adverse event (SAE), a major protocol violation, suspected drug interaction, change in health status, pathologically changed laboratory values, or noncompliance with the study drug or the visit schedule. Subjects must be discontinued if they withdraw consent, are lost to follow-up, at trial closeout, have a confirmed pregnancy, are diagnosed with gonorrhea or chlamydia or pelvic inflammatory disease during the study, have a suspected unexpected SAE related to the study drug, or have a serious problem leading to need for critical care or surgery. Subjects have a right to withdraw from the study at any time for any reason and will be informed of this right upon entering the study.

### Outcomes

The primary objective of the study is to determine the effect on cervical mucus score following delayed pill intake (by 6 hours) or after a missed pill as compared to reported daily use of norgestrel 75 mcg by measuring the difference in cervical mucus score between the day of a delayed pill or missed pill, the day after, and the day before.

The secondary objectives are as follows. To assess cervical mucus protection, we will evaluate the duration of effect of a cervical mucus score ≤4 after last pill intake of norgestrel 75 mcg after reported perfect use. Cervical mucus is considered protective when achieving a Modified Insler score ≤4 (see Box S3 in Multimedia Appendix 1). We will evaluate and compare the proportion of subjects with a protective cervical mucus score during reported daily use of norgestrel 75 mcg, during a treatment period with a delayed intake of 6 hours, and during a treatment period with a missed pill. We will evaluate and compare the ovarian activity of norgestrel 75 mcg as categorized by a modified Hoogland score (Multimedia Appendix 2) during reported perfect daily use, during a treatment period with a delayed intake of 6 hours, and during a treatment period with a missed pill. We will assess conception protection of norgestrel 75 mcg by evaluating if a protective cervical mucus score plus ovarian quiescence is maintained during reported daily use, during a treatment period with a delayed intake of 6 hours, and during a treatment period with a missed pill. We will determine plasma levonorgestrel pharmacokinetics after a single dose of norgestrel 75 mcg, at steady state, and after a delayed intake and after a missed pill. We will assess the safety of norgestrel 75 mcg taken daily for 12 weeks (SAE/AEs, bleeding patterns, abnormal blood chemistry or blood count parameters).

### Data Management and Statistical Analysis

Data will be prospectively collected and entered into a secure, centralized, electronic data capture system as soon as possible after the information is collected. Any outstanding entries must be completed immediately after the final examination.

We will analyze 4 populations of interest: (1) full-analysis population consisting of all evaluable subjects, who will be classified into 4 analysis populations depending on the question of interest: cervical mucus primary and secondary objectives, ovarian activity, and conception protection; (2) a per-protocol population composed of subjects from the full-analysis population having completed the study without major protocol violations or deviations and good compliance who will also be classified into 4 analysis populations: cervical mucus primary and secondary objectives, ovarian activity, and conception protection; (3) intent-to-treat consisting of all subjects randomized; (4) a safety population that consists of all subjects who took at least 1 dose of the study drug.

### Adverse Events

AE and SAE reporting will be in accordance with good clinical practice guidelines [14].

## Results

Our protocol was successfully approved by a central institutional review board (Advarra, Columbia, MD), received ethical approval on March 23, 2018, and registered with ClinicalTrials.gov (NCT03585712). As of January 2020, the study completed enrollment of 52 subjects across 2 sites. Analyses are pending.

## Discussion

Lapses in adherence are a common occurrence with daily dosing of any medication [15]. Studies demonstrate that, over time, contraceptive pill users have more difficulty with strict adherence [16,17]. Current clinical guidance recommends that POP users who take their pill 3 hours late should consider that a missed pill, but these recommendations are not specific to a POP containing norgestrel 75 mcg nor are they based on updated methods of monitoring pharmacodynamic endpoints [8]. Our protocol focuses on obtaining baseline pharmacodynamics of daily norgestrel 75 mcg utilizing serial monitoring plus 2 different types of adherence lapses, a delayed pill by 6 hours and a missed pill. A greater understanding of how a lapse might impact POPs and the risk for pregnancy is a critical gap in the literature that our study is designed to answer.

Our endpoints will be gathered using standard processes and by teams experienced in cervical mucus and ovarian activity monitoring, which will decrease variability in our outcome measures. Additionally, our randomized study design utilizing appropriate concealment and allocation techniques decreases the risk of bias that might occur with a nonrandomized design. Our inclusion and exclusion criteria are generalizable to the population of users who would use this pill outside of a research study except for the exclusion of individuals with a BMI >32 kg/m^2^. Ideally, pregnancy would be the endpoint to determine if these adherence lapses increase the risk for pregnancy instead of the biomarkers of pregnancy that we are monitoring. However, it would be unethical to place patients at potential risk for pregnancy, and the sample size would not be feasible. Our outcomes will be based on subject compliance with study drug and study procedures. We plan to utilize several different compliance measures including daily self-report, frequent in-person visits, and study drug counts, but we are not collecting additional objective measures of compliance in the form of frequent serum levonorgestrel levels nor are we doing daily observed pill intake as this would not be feasible. Individuals that report nonadherence or are found to be nonadherent to study drug will be discontinued from ongoing study participation. For individuals with less obvious incursions but with pharmacodynamic endpoints that appear consistent with nonadherence (ovulation and favorable cervical mucus), we can retroactively perform levonorgestrel testing on the blood samples obtained.

Unintended pregnancy is considered a major public health issue that can adversely impact an individual’s health and well-being as well as that of their community. Increased access to effective, safe methods of contraception, like increasing over-the-counter options, may help to decrease unintended pregnancy. We designed our proposed study to gain further critical information necessary to pursue over-the-counter status and to fill an important gap in the literature.

## Appendix

Multimedia Appendix 1 Supplementary materials: information regarding study outcomes, inclusion and exclusion criteria, and cervical mucus scoring.

Multimedia Appendix 2 Supplementary table: modified Hoogland score.

## Abbreviations

AE: adverse event
POP: progestin-only pills
SAE: serious adverse event
TVUS: transvaginal ultrasonography examination
