# Supplementary material for: Assessing the Pregnancy Protective Impact of Scheduled Nonadherence to a Novel Progestin-Only Pill: Protocol for a Prospective, Multicenter, Randomized, Crossover Study
Source: JMIR Res Protoc. 2021 Jun 8;10(6):e29208. doi: 10.2196/29208 (PMC8262664; doi:10.2196/29208)
Supplement: Multimedia Appendix 1 [file resprot_v10i6e29208_app1.docx]

**Box S1. Study Outcomes**

| **Primary Objective** |  |
| --- | --- |
| Change in cervical mucus score | Cervical mucus sampling on 3 consecutive days (Delayed/Missed pill periods) |
| **Secondary Objectives** |  |
| Duration of the protective effect of cervical mucus | Cervical mucus sampling on 3 consecutive days (Delayed/Missed pill periods) |
| Percentage of subjects with a protective cervical mucus score | Cervical mucus sampling at each visit |
| Ovarian activity | Ultrasound and hormones levels at each visit |
| Combination of cervical mucus score and ovarian status | Cervical mucus sampling, ultrasound, and hormone levels at each visit |
| Levonorgestrel concentrations | Plasma sampling at Day 1, Week 3, Week 5, Week 9 and on 3 consecutive days (Delayed/Missed pill periods) |

| **Box S2 Inclusion and exclusion criteria** |
| --- |
| **Inclusion Criteria** |
| - Healthy - Age 18 to 35 - Body mass index less than 32kg/m^2^ - Intact uterus and both ovaries - Regular menstrual cycles (21-35 days) - Proven ovulation (luteal phase progesterone > 3 ng/mL) - Not at risk for pregnancy during the study period - If recently but not currently pregnant, have at least one normal menstrual cycle prior to enrollment. - If recently but not currently using hormonal contraception or treatment, have at least one normal menstrual cycle prior to screening. For depot medroxyprogesterone acetate, the last injection needs to be 9 months prior to screening. - Not at risk for pregnancy during the study period |
| **Exclusion Criteria** |
| - Pregnancy - Trying to conceive or desire to conceive in the next 3 months - Currently breastfeeding, or within the last 2 months - Known Polycystic Ovarian Syndrome (PCOS) - Cancer (or past history of any carcinoma or sarcoma) - Known abnormal thyroid status, if in clinical judgment of the investigator it cannot be controlled during the study - Known hypersensitivity to the ingredients of the norgestrel or its excipients - Current acute liver disease and/or benign liver tumors - Have vaginal or cervical infection including clinical evidence of bacterial vaginosis - Evidence of abnormal cervical lesion - History of excisional or ablative treatment procedure on cervix - Undiagnosed abnormal uterine bleeding - Prior malabsorptive-type bariatric surgery - Known or suspected alcoholism or illicit drug abuse - Use of any hormonal contraception or IUD other than the study medication during the study (including emergency contraception) - Use of any medications that can interfere with the metabolism of progestin-based contraceptives (e.g CYP3A4 enzymes inducers or inhibitors, etc) - Unstable diabetes mellitus - Current participation in any other trial of an investigational medicine or participation in the past two months (or within 5 elimination half-lives for chemical entities or 2 elimination half-lives for antibodies, whichever is the longer) before screening - Abnormalities in laboratory results or TVUS performed at screening visit recognized as clinically significant by the investigator - Conditions not suitable for frequent TVUS examinations - In custody or submitted to an institution due to a judicial order - Relative or household member of the investigator's or sponsor's staff |

| **Box S3 Cervical mucus scoring** |
| --- |
| Volume not assessed |
| Viscosity  0 = thick, highly viscous, premenstrual mucus  1 = mucus of intermediate viscosity  2 = mildly viscous mucus  3 = watery, minimally viscous, mid-cycle (preovulatory) mucus |
| Spinnbarkeit  0 = <1 cm  1 = 1–4 cm  2 = 5–8 cm  3 = 9 cm or more |
| Cellularity  0 = >20 cells per high powered field (HPF) or >1000 cells per μL  1 = 11–20 cells per HPF or 501–1000 cells per μL  2 = 1–10 cells per HPF or 1–500 cells per μL  3 = 0 cells |
