# Supplementary material for: Assessing the Pregnancy Protective Impact of Scheduled Nonadherence to a Novel Progestin-Only Pill: Protocol for a Prospective, Multicenter, Randomized, Crossover Study
Source: JMIR Res Protoc. 2021 Jun 8;10(6):e29208. doi: 10.2196/29208 (PMC8262664; doi:10.2196/29208)
Supplement: Multimedia Appendix 2 [file resprot_v10i6e29208_app2.docx]

**Table S1. Modified Hoogland Score**

| Ovarian Activity Score | Activity | Follicle size / sonographic image | Estradiol (nmol/L) | Progesterone (nmol/L) | Ovarian Activity Status |
| --- | --- | --- | --- | --- | --- |
| 1 | No activity | ≤ 10 mm | Independent of E2 level | ≤ 5 | Quiescence  (score ≤ 3) |
| 2 | Potential activity | > 10 and ≤ 13 mm | Independent of E2 level | ≤ 5 | Quiescence  (score ≤ 3) |
| 3 | Non-active follicle like structure | > 13 mm | ≤0.1^a^ | ≤ 5 | Quiescence  (score ≤ 3) |
| 4 | Active follicle like structure | > 13 mm | >0.a^2^ | ≤ 5 | Ovarian activity  (Score 4 or 5) |
| 5 | Postovulatory, low progesterone (P) level | Postovulatory image^a^ | >0.1^a^ | ≤ 10 | Ovarian activity  (Score 4 or 5) |
| 6 | Postovulatory, intermediate P level | Postovulatory image^b^ | > 0.1^a^ | > 10 and ≤ 30 | Only 1 P > 10 and ≤ 30  → Ovulation abnormal luteal phase(score 6 only at one visit) |
|  |  |  |  |  | 2 consecutive P > 10 and ≤ 30  → Ovulation normal luteal phase (score 6 at two visits or score 7) |
| 7 | Postovulatory, high P level | Postovulatory image^b^ | > 0.1^a^ | > 30 | Ovulation normal luteal phase (score 6 at two visits or score 7) |

^a^~27.24 pg/mL

^b^ A postovulatory image will be defined as follows:

- Image observed after abrupt disappearance of dominant follicle OR
- Image observed after reduction in size of the leading follicle > 4 mm at two consecutive visits OR
- Haemorrhagic and cystic corpus luteum (at least as large as the leading follicle before ovulation)
